# Heart rate variability in late pregnancy: exploration of distinctive patterns in relation to maternal mental health

**DOI:** 10.1038/s41398-021-01401-y

**Published:** 2021-05-14

**Authors:** Mary C. Kimmel, Emma Fransson, Janet L. Cunningham, Emma Brann, Karen Grewen, Dario Boschiero, George P. Chrousos, Samantha Meltzer-Brody, Alkistis Skalkidou

**Affiliations:** 1 Department of Psychiatry, University of North Carolina, Chapel Hill, NC USA; 2 Department of Women’s and Children’s Health, Uppsala University, Uppsala, Sweden; 3 Department of Neurosciences, Psychiatry, Uppsala University, Uppsala, Sweden; 4 BIOTEKNA, Venice, Italy; 5 University Research Institute of Maternal and Child Health and Precision Medicine, UNESCO Chair on Adolescent Health Care, National and Kapodistrian University of Athens, Medical School, Athens, Greece

**Keywords:** Diagnostic markers, Physiology

## Abstract

Exploration of photoplethysmography (PPG), a technique that can be translated to the clinic, has the potential to assess the autonomic nervous system (ANS) through heart rate variable (HRV) in pregnant individuals. This novel study explores the complexity of mental health of individuals in a clinical sample responding to a task in late pregnancy; finding those with several types of past or current anxiety disorders, greater trait anxiety, or greater exposure to childhood traumatic events had significantly different HRV findings from the others in the cohort. Lower high frequency (HF), a measure of parasympathetic activity, was found for women who met the criteria for the history of obsessive–compulsive disorder (OCD) (*p* = 0.004) compared with women who did not meet the criteria for OCD, and for women exposed to greater than five childhood traumatic events (*p* = 0.006) compared with those exposed to four or less childhood traumatic events. Conversely higher low frequency (LF), a measure thought to be impacted by sympathetic system effects, and the LF/HF ratio was found for those meeting criteria for a panic disorder (*p* = 0.006), meeting criteria for social phobia (*p* = 0.002), had elevated trait anxiety (*p* = 0.006), or exposure to greater than five childhood traumatic events (*p* = 0.004). This study indicates further research is needed to understand the role of PPG and in assessing ANS functioning in late pregnancy. Study of the impact of lower parasympathetic functioning and higher sympathetic functioning separately and in conjunction at baseline and in relation to tasks during late pregnancy has the potential to identify individuals that require more support and direct intervention.

## Introduction

Mood and anxiety disorder episodes during pregnancy and the postpartum period are common, with 10–20% meeting full criteria for a major depressive disorder (MDD) episode^1^ and 20% meeting criteria for one or more anxiety disorders^2^. Maternal mood and anxiety disorders during pregnancy and the postpartum period are associated with negative outcomes including: preterm birth and low birth weight; increased risk of physical and mental problems; and a higher risk of suicide during and beyond the postpartum period^3–5^. Well-functioning stress systems are critical to adapt to changes during pregnancy, delivery and postpartum, and dysregulation increases risk of and results from mood and anxiety disorder episodes^6^.

An individual’s ability to react to change, adjust the stress response and then recover, has been shown as an important indicator of long-term health^7^. The optimal stress response is neither too little nor too great. The repeated need of stress responses, particularly exaggerated or underperforming responses, over time, may result in a loss of ability to respond or conversely, to respond in situations that do not require a response, both of which can be harmful and result in illness^6,7^. The concepts of allostatic load^8^, a state of homeostasis, and cacostatic load^7^, defective adaptation, provide a conceptual framework for understanding the “wear and tear” associated with stressors over time. This is a useful framework for the perinatal period as the stressors before pregnancy are then compounded by adaptations to the changes required during pregnancy, delivery, and the postpartum period. For example, women with greater trait anxiety are more likely to develop a depressive disorder postpartum^9^. Women with exposure to greater number of childhood adverse life events have an increased risk of postpartum psychiatric disorders^10^. Despite knowing that some women are at higher risk in pregnancy for perinatal mental health disorders and related negative outcomes for mother and baby based on history, new tools are needed to identify at an individual level when there is defective adaptation resulting in mental health disorders. Another important consideration is that the majority of women receive all of their care including mental health care in obstetrical settings where the mental health services are often limited by time, specialized training, and lack of providers. This further complicates identifying those individual women who require more support, providing greater need for new objective tools.

One tool that is thought to reflect emotional regulation and dysregulation during times of stress is heart rate variability (HRV), which consists of changes in the time intervals between consecutive heartbeats^11,12^. Photoplethysmography (PPG) has grown in popularity due to accessibility of use for evaluating the autonomic nervous system (ANS) with resulting measures that correspond to HRV^13–15^. For HRV measures to be a tool that can be widely utilized in perinatal women and be most clinically relevant, methods of assessment need to be conveniently implemented in maternal care settings such as obstetric offices and/or by patients at home, especially as some care has become virtual.

The nomenclature standards for HRV were initially suggested by the Task Force of the European Society of Cardiology and the North American Society of Pacing and Electrophysiology founded in 1996 and explained by Shaffer and Ginsberg (Supplement Table 5 with additional information adapted from Shaffer and Ginsberg)^11,16^. HRV is defined in the following two ways: first, time-domain measures quantify the amount of variability in measurements of interbeat intervals, the time period between heartbeats, and include the standard deviation in normal-to-normal R-R intervals (SDNN) and the root mean square of successive R-R interval differences (RMSSD). Second, frequency-domain measures estimate the distribution of absolute or relative power in frequency bands of heart period oscillations and indicate the signal energy found with a frequency band: high frequency (HF), low frequency (LF), and very low frequency (VLF). Frequency is measured in absolute power as calculated by ms squared divided by cycles per second (ms^2^/Hz); and relative power as the percentage of total HRV power and divides the absolute power for the specific frequency band by the summed absolute power of the LF and HF bands. These values are thought to reflect the dynamic relationship of systems such as the parasympathetic nervous system (PNS) and sympathetic nervous system (SNS)—but also the contribution of other systems such as the central nervous, endocrine, and respiratory systems—that control the time between heartbeats^11^. For example, the PNS predominates at rest and withdraws when greater output is needed, but may also rebound with high levels of stress and causing PNS-dominated effects^11^. Different measures are thought to reflect different components, e.g., HF reflecting vagal modulation of heart rate^11^. Although LF and the LF/HF ratio are debated with regards to their meaning and importance and are not supposed to be considered only a measure of the SNS; they have increasingly been studied and provide different information than the HF band^11,17^.

HRV measures have been compared with other traditional compound metrics of cacostatic load and comparably indicate well-being, homeostasis, and dysregulation of stress responses^18^. Low HRV has been associated with greater mortality^19^. In non-pregnant populations, low or excessive as well as unstable HF are associated with psychiatric diagnoses^12,20,21^. In addition to HF, other HRV measures have also been associated with anxiety disorders. For example, total power, LF, SDNN, and the LF/HF ratio have been found to differ between those with higher anxiety, posttraumatic stress disorder (PTSD) or panic disorder and those with lower anxiety, without panic, and not having PTSD^22–27^. Several studies have gone further to study the links between HRV and prefrontal cortex (PFC) function^12,28–30^. Important as both increased and decreased PFC functioning are linked to psychiatric diagnoses including MDD, bipolar disorder, obsessive–compulsive disorder (OCD), PTSD, and social phobia^20,31–34^.

During pregnancy numerous ANS adaptations are required such as sympathetic activation compensatory for systemic vasodilation and decrease in mean arterial pressure^35^. Adaptation is required, as pregnancy progresses, to maintain eustasis, i.e., healthy homeostasis, reflected in changes in HRV, including increasing attenuation of stress responsiveness^36–41^. Although HRV has been studied in pregnancy^39,41–45^, HRV has not been studied in pregnant women with mood and different forms of anxiety disorders, which is necessary to understand if the ANS adaptations are different for these women. In the present study, HRV profiles obtained during gestational week 38 of the third trimester of pregnancy from PPG before and after exposure to a working memory task, a standardized task that can be administered in a maternal care clinic, were explored in a group of women whose psychiatric diagnoses were characterized.

## Methods

### Subjects

The current study was undertaken as part of the Biology, Affect, Stress, Imaging and Cognition (BASIC) cohort^46^, a population-based study at Uppsala University Hospital, Sweden. The primary aim of the BASIC cohort is to investigate correlates of affective symptoms during pregnancy and after childbirth. Women who register for the routine ultrasound examination at the hospital around gestational week 17 were asked to participate. Exclusion criteria were age <18 years, not being able to adequately communicate in Swedish, protected identity, blood borne infectious diseases, and non-viable pregnancies. The BASIC study collected data through web surveys sent out at the time of consent (around gestational week 17), at gestational weeks 32, as well as at 6 weeks, 6 months, and 12 months postpartum. An example of the surveys is the Edinburgh Postnatal Depression Scale (EPDS)^47^.

Participants were invited to the research laboratory of the Department of Obstetrics and Gynecology at approximately gestational week 38 for an in-person study visit beyond the surveys. Starting in 2014, the substudy in-person visits also included PPG before and after the working memory task, the Wechsler Digit Span Test (DST)^48^. The cohort was enriched with subjects who either had EPDS scores above 12 or scores below 6 to be able to compare subjects likely to have depression and those more likely to not have depression. We also enriched the cohort with women taking SSRIs, regardless of their current level of depressive or anxiety symptoms to reflect real-world care of perinatal women and supported by findings from the literature that have shown differences in several biological measures in those on SSRI treatment compared with untreated depressed women^49^. Between January 2010 and December 2018, 715 invitations were sent out for pregnancy test sessions with 349 pregnancy test sessions completed, half after 2014 (48.8% participation rate including half of those with elevated EPDS scores at 32 weeks)^46^.

The Mini-International Neuropsychiatric Interview (MINI) was administered in order to characterize women by current or past episodes of MDD, bipolar disorder (presence of a manic or hypomanic episode ever), or panic and past history of anxiety disorders agoraphobia, OCD, generalized anxiety disorder (GAD), and social phobia^50,51^. Participants filled out the EPDS and the State-Trait Anxiety Inventory for Adults (STAI), the latter previously used in pregnancy^52,53^. From EPDS scores at the 38-week visit a cutoff of >12 was utilized to determine those more likely to have a depressive disorder^47^. Three questions (EPDS 3 A) can be utilized to assess anxiety, and cutoff scores of above 4 and 6 have been validated with a score of 5 or greater recommended^54,55^. We used a cutoff score of 40 or greater for the STAI-Trait subscale for elevated trait anxiety and cutoff of 12 or greater for the state (six items) subscale^53^. The STAI cutoff score above 40 is not only validated in pregnancy but as a predictor of postpartum anxiety and mood states; so likely a measure of both current and ongoing anxiety^53^. The Life Incidence of Traumatic Events (LITE) was given to assess number of exposures to traumatic events limited to occurring during childhood (up to 18 years of age)^56^. Each individual was also characterized as to whether they had an elevated number, above 5, of traumatic events in childhood (75th percentile). Childhood trauma was the focus given the association of maternal childhood trauma with perinatal mood and anxiety disorders^57–59^. The LITE has been studied in its ability to assess for exposure to childhood traumas so that the cumulative effects can be studied in relation to other factors and outcomes^60^. While the LITE does not assess PTSD symptoms, it has been found to be correlated with posttraumatic stress, depression, and anxiety^60^. Also included in the analyses were treatment with SSRIs and personal characteristics such as age and BMI. Participants were determined to have severe delivery fear based on either self-report of being “terrified” of delivery or based on medical record notes of a scheduled visit to a fear of birth clinic.

The HRV portion of the visit was done at the end of the visit after individuals completed demographic information and answered questions such medical history including current medication usage. HRV was recorded using a PPG transducer (model PPG stress flow, provided by BioTekna, Marcon (Italy))^61^. with one electrode placed on each index finger. Studies have found this method of measuring pulse rate variability (PRV) to have sufficient accuracy for estimating HRV^62,63^. The participants were told to try to relax but were allowed to see their heart rate on the computer screen while recording. Two measurements, of 5 minutes each, were conducted; the second one following the DST. Both time-domain measures (RMSSD and SDNN) and frequency-domain measures (HF power, LF power, very low frequency or VLF power and Total power) were calculated by the device from BioTekna. There were no restrictions in food and caffeine intake or tobacco use before the visit.

A group of healthy non-pregnant controls aged 22–42 years with BMI within the range of 20–29 kg/m^2^, with parity <4 and with no systemic disease or current psychiatric conditions were also invited to complete the same assessments as in the BASIC study including having HRV measures recorded. The healthy controls had never or not during the past two years given birth and had completed breastfeeding at least 3 months before. The women had regular periods, were using combined contraceptives, intrauterine device, or no contraceptives at all, and could not be suffering from premenstrual syndrome. To standardize for the menstrual cycle, the healthy non-pregnant participants were asked to come for the assessment on day 16–26 (luteal phase) in the menstrual cycle.

### Ethical considerations

The study protocol has been approved by the Regional Ethical Review Board in Uppsala, Sweden (Dnr 2009/171) and conducted in accordance with the Declaration of Helsinki. Written informed consent was obtained from all participants to participate in the BASIC study, as well as when participating in the substudy prior to any testing.

### Statistical analyses

To calculate percent change in HRV measures, each person’s measure from before the task was subtracted from the value after the task and then divided by the measure before the task. For the STAI and EPDS results, missing data were imputed if only one response was missing.

To test the associations between psychiatric diagnoses including MDD, panic disorder, GAD, OCD, bipolar disorder, social phobia, and agoraphobia (yes/no), symptoms (over/under cutoff), BMI, age on the one hand and HRV variables on the other, linear regressions were applied. Subjects were grouped based on the cutoffs into categorical variables that were included in the regression analyses. Individuals who met MINI criteria for a diagnosis were compared against all others who did not meet criteria for that diagnosis regardless of other diagnoses for which they may have met criteria. HRV variables were plotted in histograms to test normality. For LF/HF and RMSSD, which were assessed as non-normally distributed, Mann–Whitney non-parametric testing was applied.

Fisher’s exact test was applied for BMI and age in relation to the psychiatric diagnoses, fear of childbirth, STAI results, EPDS results, and exposure to trauma.

To adjust for multiple testing with regards to the regression analyses, Bonferroni correction was applied to consider the multiple comparisons occurring. Those with *p* values <0.00625 were considered significant given eight HRV measures. The data were analyzed using the Statistical Package for the Social Sciences (SPSS) version 26.0 (IBM SPSS, Armonk, NY).

## Results

### Demographics

One hundred and twenty-six subjects participated in the third trimester visit that included the HRV measures. The average age of subjects was 31.6 years (s.d. of 4.4) with a range of 21–43 years. One hundred and fourteen subjects, or 93%, reported having been born in Scandinavia. Other subjects reported evenly between having been born in Europe outside of Scandinavia, Asia, Africa, or did not report where they were born. Almost 80% had a university level education and 65% identified as working full-time at the gestational week 17. By gestational week 32, 38% were working full-time, 25% working part-time, and 20% taking leave due to the pregnancy. The average BMI before pregnancy, based on their self-reported weight by the participants was 24.3 kg/m^2^ (s.d. 4.4 kg/m^2^) with a range of 17.7 to 39.8 kg/m^2^.

### Prevalence of mood and anxiety diagnoses and respective risk factors

There were 42% who did not meet criteria for any psychiatric diagnosis from the MINI Of these who did not meet criteria for a psychiatric diagnosis, 13% had past exposure to greater than five childhood traumatic events, 32% did not fill out the LITE, one was taking a SSRI, and three had EPDS scores above 12. There were 45% with a history of MDD, including 26% who had a diagnosis of MDD as their only diagnosis. Panic disorder was the next largest diagnosis with 15%, followed by 12% with a GAD. Despite the large percent with history of depression or anxiety disorders, only 6.4% were taking an SSRI at 17 and/or 32 weeks’ gestation (medication information was missing for 24 women). Table 1 shows the distribution of diagnoses by individual.

**Table 1:** MINI diagnoses and co-morbidities.

Table shows the number of women in the BASIC cohort included in these analyses with a diagnosis or combination of diagnoses as determined by the MINI The final column shows the number with each diagnosis. Note that in the “None” group, some individuals did not have LITE results, had greater than five childhood traumatic events, or had elevated EPDS scores. One was taking an SSRI.

The average number of childhood traumatic events was 3.7 (s.d. 2.8), ranging from no childhood traumatic events to 11 childhood traumatic events for one person.

The average score on the STAI-Trait was 36.7 (s.d. 9.8). The average score on the STAI-State was 9.5 (s.d. 2.8) with a range of 6–19. The average score on the EPDS during the visit was 6.2 (s.d. 4.5) with range of 0–18. There were 11% of subjects who had an elevated total EPDS score at the 38-week visit, but also an additional 15%, who despite scoring 12 or below, had an elevated score on the three anxiety questions of the EPDS and/or a positive screen for suicidal or self-harm thoughts.

### Mean heart rate variability measures in the third trimester of pregnancy

#### HRV measures in comparison with other studies in pregnancy

The average and standard deviation of HRV measures from this study were similar to others reported in the literature for women in the third trimester (see Table 2). In response to the stressor, across all individuals the mean HF and RMSSD values showed increases from before and after the tasks while VLF and HR mean values were lower after the tasks.

**Table 2:** Heart rate variability measures of non-pregnant and in the third trimester across studies in the field.

	BASIC									Stein (24 hr) mean ± s.d.	
	Pregnant			Healthy non-pregnant controls*						Late pregnancy	Non-pregnant
	Pre-stressor, mean ± s.d., range	Post-stressor, mean ± s.d., range	Percent change, mean (±s.d.)	Mean ± s.d. (range)	Mizuno, mean ± s.d. (range)	Gandhi, mean ± s.d.	Braeken, mean ± s.d.	Maser mean ± s.d.	Logan mean ± s.d.		
*N*	125	122	125	106	45	30	129	30	15	8	
HR (bpm)	87 ± 12, (59–119)	84 ± 11, (58–117)	−0.037, (0.058)	68.7 ± 10, (49–96)	79 ± 10	85 ± 3	89 ± 10	89 ± 8	87 ± 10	87 ± 8	76 ± 4
HF percent	46.5 ± 18.2 (10.1–87.3)	47.7 ± 18.7 (9.0–86.5)	+0.105 (0.459)	46.6 ± 19 (10–87)							
HF power (ln)	5.7 ± 1.3, (2.0–8.9)	6.0 ± 1.1, (3.1–8.9)	+0.059, (0.178)	7.2 ± 1.3, (4–11)	5.0 (3.9–6.1)	5.3 ± 5.2	5.3 ± 1.2	5.7 ± 5.8	6.2 ± 6.5	6.5 ± 0.6	7.0 ± 0.6
LF power (ln)	5.9 ± 0.9 (3.1–8.3)	6.1 ± 0.8 (4.1–8.2)	+0.034 (0.124)	7.4 ± 0.8 (4–10)		6.0 ± 5.9		5.6 ± 5.6	5.3 ± 5.0	6.9 ± 0.3	7.5 ± 0.3
LF/HF ratio	1.7 ± 1.6 (0.1–8.9)	1.6 ± 1.6 (0.2–10.2)	+0.156 (0.743)	1.7 ± 1.6 (0–9)				1.2 ± 0.8	0.7 ± 13.2		
Total power (ln)	7.8 ± 0.8 (5.7–9.7)	7.8 ± 0.8 (5.7–9.7)	+0.003 (0.078)	8.6 ± 0.9 (7–11)						9.5 ± 0.5	10.2 ± 0.4
VLF power (ln)	7.2 ± 0.8 (5.3–9.6)	7.2 ± 0.9 (5.5–9.4)	−0.006 (0.106)	7.3 ± 0.8 (6–9)	6.5 (5.8–7.2)	6.8 ± 6.6				7.5 ± 0.5	8.1 ± 0.2
RMSSD	30.2 ± 24 (8–224)	32.2 ± 17.9 (9–104)	+0.195 (0.481)	73.5 ± 64.3 (13–335)		22.2 ± 19			23.7 ± 17	39 ± 17	55 ± 15
SDNN	53.5 ± 22 (18–153)	53.4 ± 20.5 (22–131)	+0.045 (0.321)	83.1 ± 47.8 (27–279)		27.9 ± 12			23.7 ± 17	127 ± 32	169 ± 25

Table shows mean with standard deviation and ranges for each HRV measure for pregnant women in the BASIC cohort and for a group of non-pregnant controls, also from Sweden. Non-pregnant controls had not given birth within the past 2 years, if breastfeeding stopped over 3 months ago. The table also includes means and standard deviations from HRV measures from pregnant women in studies in the literature.

#### HRV measures in pregnancy in comparison with non-pregnant subjects

Heart rate in pregnant women versus non-pregnant women from Uppsala was elevated as shown in Table 2, whereas HF power, LF power, RMSSD, and SDNN was lower.

### Heart rate variability measures by mood and anxiety diagnoses and their risk factors

Table 3 presents the overall patterns of HRV measures per psychiatric diagnosis, scores on the EPDS, states and traits of anxiety from the STAI, severe fear of childbirth, whether taking a SSRI, and those exposed to a greater number of childhood traumatic events. Those with *p* values <0.00625 were considered significant given eight HRV measures (dark red and blue in Table 3). *P* values <0.05 and <0.10 were also noted in Table 3 as lighter reds and blues. Supplement Table 1 through 4 contain tables with more details on findings.

**Table 3:** HRV patterns across different MINI diagnoses and other characteristics during third-trimester pregnancy visit.

^1^Any fear of birth was not statistically significant. There were 43 individuals with any fear and 82 not reporting fear. Fear of childbirth included fear of C-section, fear of vaginal delivery, and severe fear for any of the above. BMI was also included in the model for fear of childbirth only since it was only significantly associated with this variable.

^2^Age was included in the model for major depression since it was only significantly associated with major depression.

After correction for multiple comparisons, at least one HRV measure differed between those with a specific psychiatric diagnosis (past or current) and all others (those not meeting criteria for that specific psychiatric diagnosis) including: panic disorder, social phobia, and OCD. As shown in Table 3, Lower HF, a measure of parasympathetic activity, was found for women who met criteria for history of OCD (*p* = 0.004) compared with women who did not meet criteria for OCD, and for women exposed to greater than five childhood traumatic events (*p* = 0.006) compared with those exposed to four or less childhood traumatic events. Conversely, higher LF, a measure, thought to be impacted by sympathetic system effects, and the LF/HF ratio were found for those meeting criteria for a panic disorder (*p* = 0.006), meeting criteria for social phobia (*p* = 0.002), elevated trait anxiety (*p* = 0.006), and exposure to greater than five childhood traumatic events (*p* = 0.004).

In terms of current elevated distress symptoms, as measured by the EPDS at the 38-week visit, including separately the EPDS 3-A, no findings were significant after controlling for multiple comparisons. When all with one or more MINI diagnoses, regardless of type of diagnosis, were included in one group compared with those with no MINI diagnoses there were no differences that remained statistically significant after consideration for multiple comparison.

Even after adjusting for multiple comparisons, change in VLF from before and after the two tasks of those with SSRI use at 17 and/or 32 weeks in pregnancy was different than those not taking SSRI.

Although BMI and age were both associated with differences HRV measures, BMI was not associated to the diagnoses or related variables with exception of the fear of childbirth condition. Almost half of those who were overweight or obese had severe fear of delivery as compared to being only 28% of those with lower fear of delivery (Fisher’s exact two-sided *p* value 0.046). Age was only a factor for a positive screening on the MINI for MDD; those 35 and older making up 35% of those with a diagnosis of MDD versus 16% of those without (Fisher’s exact two-sided *p* value 0.021). Results reported in the tables for fear of childbirth are adjusted for BMI and MDD is adjusted for age.

## Discussion

In this study, HRV measures captured from PPG before and after exposure to DST were explored in a clinical sample of pregnant women at gestational week 38. Women were characterized in terms of current and past depression, bipolar manic, and anxiety disorder episodes and risk factors for perinatal mood and anxiety disorders including trait anxiety and exposure to traumatic events during childhood. This study explores the complexity of mental health of a population-based sample of individuals, representing those seen in maternal care settings, showing the difficulty of defining cases and controls given the number of different diagnoses, risk factors, and co-morbidities, which reflects the situation in clinical settings. The primary finding was that those who meet criteria for several different anxiety disorders as well as women with higher trait anxiety or greater exposure to childhood traumatic events had evidence of ANS alterations compared with others in late pregnancy. Anxiety may be associated with greater ANS alterations than depression, or depression in this sample may be more heterogenous, or not as severe; the anxiety findings are particularly relevant owing to one in five women having an anxiety disorder in pregnancy, whereas mood disorder episodes are more prevalent in the postpartum period^1,2^<./p> Pregnancy is a known stressor with effects that extend beyond the perinatal period, with a well-known example being the emergence of diabetes and hypertension during pregnancy that are associated with increased cardiovascular risks later in life^35,64,65^. Mean HRV values across all pregnant women were lower compared with non-pregnant women and consistent with other studies in pregnant women, regardless of use of PPG or ECG for measurement^39,41–45^.

### Unique ANS alterations in the late pregnancy based on mental health history and risk

The findings from this study of a population-based maternal care clinically relevant sample support the notion that women who meet criteria for certain diagnoses or risk factors have lower PNS activity, whereas individuals meeting criteria for other diagnoses or risk factors show greater SNS activity. Interestingly, those exposed to more childhood trauma events had HRV measures reflecting both lower PNS and higher SNS. Although similar findings have been found in non-pregnant individuals (i.e., relative imbalance of HF and LF with social phobia^66^; lower HF; and increased LF/HF ratio with PTSD^24–26^), this is the first time pregnant women with anxiety disorders were shown to have distinct HRV patterns. Those with OCD and those with exposure to childhood traumatic events had similar alterations in ANS functioning, supported by literature showing childhood trauma exposure has been associated with greater obsessive–compulsive symptoms, particularly in females^67^. Obsessive–compulsive symptoms are more commonly seen in perinatal patients^68,69^. Further study is needed to determine whether this increase in symptoms associates with ANS alterations in the perinatal period. Women with panic disorder in late pregnancy in the current study had higher LF, a slightly different finding than reported by Zhang et al.^27^ in non-pregnant individuals with panic disorder, who had alteration in the ratio of HF and LF. In this study, SSRI users exhibited less change in VLF between before and after completing the memory task, compared with those not taking SSRIs. VLF is a measure that includes multiple components including the renin–angiotensin system and also SSRI use may indicate severity of mood and anxiety disorder^70^. The literature supports the importance of considering SSRIs in relation to HRV alterations, but also the contractions and complexity. HRV both predicted response to antidepressant medication, and did not respond to antidepressant treatment^71,72^. The results of this study, whereas preliminary, suggest that particularly in late pregnancy, the ANS alterations observed for different groups of psychiatric disorders are in some cases similar and in some slightly different than those observed outside the perinatal period; the impact of these ANS alterations needs to be further studied with regards to outcomes for mother and child.

### Strengths, limitations, and future directions

One strength of this study is a population-based sample with comorbid conditions that reflects clinical settings. Table 1 shows the complexity of such samples, where many individuals have co-morbidities or other risk factors. This was also true for those without a MINI diagnosis. By comparing each group based on those that met the criteria versus those that did not meet the criteria, we have addressed each diagnosis separately, regardless of other diagnoses. However, it will be important for future studies to recruit even greater numbers with single disorders and risk factors in order to better describe distinct HRV alterations. In addition, as methods of diagnosis are also dependent of self-reports some that do not meet full criteria may still have symptoms comparable to those that met criteria by the MINI for diagnosis. A dimensional approach may be important given the complexity of clinical populations.

The study was limited in power by the sample size despite being larger than previous studies of HRV in pregnancy. Some true associations may have been missed owing to the sample size. It is possible that some significant findings may be due to chance due to multiple measures and comparisons. We nevertheless also controlled for multiple comparisons and have only discussed the most robust findings. Although GAD in a non-pregnant population has been associated with differences in HF^73^, women with GAD in this study did not show significantly different HRV values. Although HF was significantly lower in non-pregnant individuals with GAD, all pregnant women had lower HF than non-pregnant women and so larger sample sizes may be needed to reveal alterations in pregnant women with GAD versus pregnant women without history of GAD. GAD in this patient population might also be more heterogenous, and a larger sample size would also further enable subdivision into more homogenous subgroups of generalized anxiety. Similarly, no robust RMSSD findings were found despite the robust HF findings; RMSSD and HF are thought to represent PNS activity from the vagus nerve on HRV and are usually correlated^11^. Given the respiratory changes, particularly in the third trimester, differences between RMSSD and HF may be exacerbated in late pregnancy. Wang and Huang note, RMSSD may not correlate with HF power given different impacts of respiration in general;^74^ more information is needed comparing RMSSD and HF in late pregnancy. Logan et al.^45^ found opposite directions in HF and RMSSD pre and post a stretching exercise further supporting that respiration is an important consideration. A larger sample size in future studies would be needed, to both validate our findings, assess if additional findings become apparent, and further study the use of PPG in characterizing distinct psychiatric conditions. However, even with the sample size limitations there were some groups that had robust alterations compared to those without those risk factors or diagnoses.

This study demonstrated that PPG has not only potential for assessment of ANS functioning but also requires further study to continue to improve its use as a biomarker in the field of perinatal mental health. Several studies have compared HRV measured from PPG as compared with other methods and found good agreement between methods^62,63^. PPG can be captured by mechanisms that could be utilized by patients in their natural environments and during day-to-day activities^62,63^. Some differences though, especially in response to a physical stressor, have been identified and merit further investigation^75,76^. Yuda et al.^76^ suggest PPG may be considered as its own biomarker, representing ANS activity and its impacts on pulse conduction. Further studies comparing findings from the behavioral laboratory with ECG in relation to PPG in maternal care settings and patient’s homes is warranted.

The future role of HRV measurement using PPG during pregnancy, in particular for those with anxiety disorders, trait anxiety, and greater exposure to childhood traumas, depends on several factors. First, the methods must be standardized and a range of normal values established. This will require inclusion of pregnant and non-pregnant women by psychiatric conditions such as by primary disorders alone and with co-morbidities. Given the consistency between HRV values during pregnancy in this and others studies, it may be possible to identify normal ranges of values. Normal values for HRV measures may need to be adjusted for factors including age and different hormonal states across the perinatal period^77,78^. As an example for the latter from the literature, trait anxiety was found to be associated with greater decrease in HF and greater HF reactivity in the third trimester; along with greater increase in VLF compared with the second trimester^41,42^. This discrepancy requires further exploration; it may reflect HRV differences between time points in the perinatal period but may also be due to methodological differences, as HRV in one study was measured during a stressor, whereas in the other HRV was taken at rest.

Second, longitudinal studies that target recruitment of greater number of women with social phobia, panic disorder, OCD, trait anxiety, and exposure to a number of childhood traumas are also needed. Larger longitudinal studies will allow study of changes in each individual across the perinatal period. Larger longitudinal studies would also improve our understanding of the use of the DST as a tool for testing memory in relation to biomarkers, but also as a possible evidence-based stressor. Memory deficit is a complaint of many women that increases during the perinatal period but also is more associated with depression and anxiety disorders^79,80^. There is evidence that women may utilize different parts of the brain to accomplish working memory tasks during different time points in pregnancy; and these different processes may result in women with anxiety having more stress in navigating the task, particularly in late pregnancy^81^. Another example where a longitudinal study would be beneficial is in understanding the finding with regards to SSRI users exhibiting less change in VLF between before and after completing the memory task, compared with those not taking SSRIs. Longitudinal assessments of women who start, maintain, or stop SSRIs are needed to better understand how SSRIs may impact on the ANS. Longitudinal assessments of PPG measures would allow for comparison not only between individuals but within individuals with assess the changing balance in ANS functioning and when imbalance first presents. Similarly, longitudinal studies would also allow for comparison of the same subjects in terms of episodes of exacerbations of psychiatric conditions while pregnant and non-pregnant beyond the perinatal period. Further, they would also improve understanding of the association between ANS alterations in late pregnancy on maternal and child outcomes. Study of the impact of lower PNS functioning and higher SNS functioning separately and in conjunction at baseline and in relation to tasks during late pregnancy has the potential to identify dyads that require more support and to direct intervention.

## Conclusions

This novel study explores the complexity of mental health disorders in women during late pregnancy, in relation to ANS functioning. The primary finding was that those who meet criteria for several different anxiety disorders as well as women with higher trait anxiety or greater exposure to childhood traumatic events had evidence of HRV alterations as measured by PPG compared with others in late pregnancy. Some HRV measures may point towards lower PNS activity in women with certain diagnoses or risk factors, others towards a shift in the balance of systems maintaining HRV, and still others towards an increase in SNS and other heightened stress responses. Continued research should be encouraged in the direction of determining the normal range of values for HRV measured by PPG. HRV is thought to reflect ANS and CNS functioning and larger studies may provide new information on ANS and CNS functioning in late pregnancy. PPG could eventually provide a tool that can be utilized in maternal care settings after larger studies further our understanding of how we can use each perinatal woman’s ANS reaction to different tasks and stressors as an intermediate phenotype, to identify high-risk individuals in need of extra support.

## Supplementary information

Supplemental Material
