# Supplementary material for: Heart rate variability in late pregnancy: exploration of distinctive patterns in relation to maternal mental health
Source: Transl Psychiatry. 2021 May 14;11:286. doi: 10.1038/s41398-021-01401-y (PMC8119957; doi:10.1038/s41398-021-01401-y)
Supplement: Supplementary file 1 — Supplemental Material [file 41398_2021_1401_MOESM1_ESM.docx]

**SUPPLEMENT Table 1: EPDS Scores and General Presence or Absence of Diagnoses, Co-morbidity and HRV Patterns**

| **Relation to Stressor** | **EPDS at 32 weeks**  **(=/>12)** | | **SSRI during week 17 or 32** | | **One or More**  **Mood or Anxiety Diagnoses**  **(1+)** | | **Two or More**  **Mood or Anxiety**  **Diagnoses**  **(2+)** | | **EPDS at Visit** | | | |
| --- | --- | --- | --- | --- | --- | --- | --- | --- | --- | --- | --- | --- |
|  |  |  |  |  |  |  |  |  | **Total (>12)** | | **3A (5+)** | |
| N (%) | 20.2 | | 6.9 | | 52.1 | | 18.8 | | 10.9 | | 15.1 | |
| **HF (P= Power, %= Percentage)/Vagal Modulation** | | | | | | | | | | | | |
|  | P | % | P | % | P | % | P | % | P | % | P | % |
| Before | 5.6  (0.259) | 43.8  (0.163) | (0.979) | (0.648) | (0.204) | -7.771(0.023) | (0.227) | -7.842 (0.070) | (0.387) | -10.886(0.049) | -0.754(0.029) | -8.974(0.066) |
| After | 5.9  (0.938) | 46.1  (0.431) | (0.735) | (0.191) | (0.241) | (0.194) | (0.323) | (0.184) | (0.911) | (0.360) | (0.172) | (0.470) |
| % Change | +0.046  (0.129) | +0.149  (0.375) | (0.879) | (0.374) | (0.747) | 0.152(0.082) | (0.167) | (0.120) | (0.326) | 0.249 (0.090) | 0.084(0.081) | 0.277(0.026) |
| **LF (Power)/Sympathetic and Some Parasympathetic** | | | | | | | | | | | | |
| Before | 5.9 (0.727) | | (0.714) | | (0.764) | | (0.977) | | (0.681) | | (0.165) | |
| After | 6.1 (0.454) | | (0.347) | | (0.778) | | (0.986) | | (0.306) | | (0.233) | |
| % Change | +0.046 (0.382) | | (0.477) | | (0.473) | | (0.898) | | (0.754) | | (0.618) | |
| **LFHF Ratio/Sympathetic and Parasympathetic Ratio** | | | | | | | | | | | | |
| Before | 1.8 (0.555) | | (0.529) | | 0.538(0.077) | | (0.116) | | (0.422) | | (0.218) | |
| After | 1.7 (0.579) | | (0.213) | | (0.653) | | (0.328) | | (0.909) | | (0.642) | |
| % Change | +0.070 (0.283) | | (0.280) | | (0.244) | | (0.354) | | (0.377) | | (0.249) | |
| **VLF/Sympathetic and Renin-Angiotensin** | | | | | | | | | | | | |
| Before | 7.3 (0.291) | | (0.158) | | (0.294) | | (0.716) | | (0.174) | | (0.783) | |
| After | 7.1 (0.950) | | (0.231) | | (0.460) | | (0.759) | | (0.377) | | (0.355) | |
| % Change | -0.021 (0.176) | | -0.115 (0.006) | | -0.037(0.066) | | (0.695) | | (0.740) | | (0.431) | |
| **Total Power/Overall Autonomic Nervous System Activity** | | | | | | | | | | | | |
| Before | 7.8 (0.946) | | (0.458) | | (0.899) | | (0.779) | | (0.524) | | (0.245) | |
| After | 7.8 (0.994) | | (0.306) | | (0.385) | | (0.730) | | (0.519) | | (0.144) | |
| % Change | +0.002 (0.897) | | -0.070 (0.026) | | (0.140) | | (0.719) | | (0.947) | | (0677) | |
| **RMSSD/Parasympathetic** | | | | | | | | | | | | |
| Before | 26.9 (0.193) | | (0.568) | | (0.183) | | (0.509) | | (0.286) | | -12.263 (0.060) | |
| After | 32.0 (0.919) | | (0.746) | | (0.344) | | (0.647) | | (0.973) | | -8.831 (0.069) | |
| % Change | 0.276(0.113) | | (0.764) | | (0.919) | | (0.205) | | 0.377 (0.013) | | 0.217 (0.096) | |
| **SDNN/ Overall Autonomic Nervous System Activity** | | | | | | | | | | | | |
| Before | 52.8 (0.768) | | (0.866) | | (0.964) | | (0.646) | | (0.990) | | (0.226) | |
| After | 52.9 (0.821) | | (0.201) | | (0.426) | | (0.878) | | (0.546) | | (0.149) | |
| % Change | +0.040 (0.884) | | -0.266 (0.041) | | (0.120) | | (0.471) | | (0.721) | | (0.595) | |
| **Heart Rate (bpm)** | | | | | | | | | | | | |
| Before | 88 (0.478) | | (0.528) | | (0.745) | | 5.119 (0.084) | | (0.890) | | 7.844 (0.021) | |
| After | 84 (0.959) | | (0.275) | | (0.984) | | (0.157) | | (0.680) | | 5.871 (0.058) | |
| % Change | -0.046 (0.150) | | (0.306) | | (0.286) | | (0.114) | | (0.710) | | (0.298) | |

**SUPPLEMENT Table 2: Depression, Bipolar Mania, and Suicidal/Self-Harm and HRV Patterns**

| **Relation to Stressor** | **Major Dep*** | | **Bipolar Mania** | | **SI/Self-Harm** | |
| --- | --- | --- | --- | --- | --- | --- |
| N (%) | 44.6 | | 5.4 | | 13.9 | |
| **HF (P= Power, %= Percentage)/Vagal Modulation** | | | | | | |
|  | P | % | P | % | P | % |
| Before | (0.336) | (0.185) | (0.653) | (0.149) | (0.522) | (0.160) |
| After | (0.508) | (0.563) | (0.304) | (0.279) | (0.793) | (0.797) |
| % Change | (0.538) | 0.150(0.093) | (0.595) | (0.808) | (0.542) | 0.210(0.093) |
| **LF (Power)/Sympathetic and Some Parasympathetic** | | | | | | |
| Before | (0.917) | | (0.459) | | (0.718) | |
| After | (0.759) | | (0.863) | | (0.562) | |
| % Change | (0.917) | | (0.276) | | (0.846) | |
| **LFHF Ratio/Sympathetic and Parasympathetic Ratio** | | | | | | |
| Before | (0.309) | | 1.153 (0.092) | | (0.276) | |
| After | (0.907) | | 1.457 (0.028) | | (0.927) | |
| % Change | (0.403) | | (0.895) | | (0.187) | |
| **VLF/Sympathetic and Renin-Angiotensin** | | | | | | |
| Before | (0.685) | | (0.545) | | (0.862) | |
| After | (0.319) | | (0.123) | | (0.994) | |
| % Change | -0.037 (0.065) | | (0.327) | | (0.746) | |
| **Total Power/Overall Autonomic Nervous System Activity** | | | | | | |
| Before | (0.759) | | (0.687) | | (0.518) | |
| After | (0.275) | | (0.209) | | (0.940) | |
| % Change | (0.233) | | (0.330) | | (0.498) | |
| **RMSSD/Parasympathetic** | | | | | | |
| Before | (0.373) | | (0.891) | | (0.514) | |
| After | (0.559) | | (0.787) | | (0.528) | |
| % Change | (0.673) | | (0.532) | | (0.357) | |
| **SDNN/ Overall Autonomic Nervous System Activity** | | | | | | |
| Before | (0.833) | | (0.792) | | (0.341) | |
| After | (0.256) | | (0.280) | | (0.917) | |
| % Change | (0.191) | | (0.232) | | (0.477) | |
| **Heart Rate (bpm)** | | | | | | |
| Before | (0.416) | | (0.756) | | (0.578) | |
| After | (0.635) | | (0.662) | | (0.178) | |
| % Change | (0.282) | | (0.893) | | (0.292) | |

*Results for Major Depression are from a model with age included. Age was only significantly associated with Major Depression of the mental health variables.

| **Relation to Stressor** | **Panic Disorder** | | **Agoraphobia** | | **Social Phobia** | | **OCD** | | **GAD** | | |
| --- | --- | --- | --- | --- | --- | --- | --- | --- | --- | --- | --- |
| N (%) | 14.6 | | 5.2 | | 4.3 | | 5.3 | | 12.3 | | |
| **HF (P= Power, %= Percentage)/Vagal Modulation** | | | | | | | | | | | |
|  | P | % | P | % | P | % | P | % | P | | % |
| Before | (0.289) | (0.160) | (0.608) | (0.890) | (0.128) | -17.266  (0.036) | -1.543  (0.004) | -17.534  (0.021) | (0.518) | (0.904) | |
| After | (0.656) | -9.990  (0.058) | (0.183) | (0.541) | (0.214) | (0.304) | -1.264  (0.006) | -16.286  (0.035) | (0.748) | (0.332) | |
| % Change | (0.356) | (0.588) | (0.333) | (0.283) | (0.241) | 0.538  (0.011) | 0.186  (0.013) | (0.563) | (0.142) | (0.531) | |
| **LF (Power)/Sympathetic and Some Parasympathetic** | | | | | | | | | | | |
| Before | 0.705(0.006) | | (0.709) | | (0.941) | | -0.723(0.067) | | (0.297) | | |
| After | 0.557(0.016) | | (0.731) | | (0.548) | | (0.123) | | (0.591) | | |
| % Change | (0.129) | | (0.391) | | (0.540) | | (0.155) | | (0.365) | | |
| **LFHF Ratio/Sympathetic and Parasympathetic Ratio** | | | | | | | | | | | |
| Before | (0.217) | | (0.342) | | 2.221(0.002) | | 1.561(0.021) | | (0.419) | | |
| After | (0.250) | | (0.692) | | (0.372) | | 1.129(0.088) | | (0.196) | | |
| % Change | (0.878) | | (0.901) | | (0.208) | | (0.586) | | (0.264) | | |
| **VLF/Sympathetic and Renin-Angiotensin** | | | | | | | | | | | |
| Before | 0.554(0.017) | | (0.354) | | -0.801(0.035) | | (0.199) | | (0.647) | | |
| After | 0.608(0.018) | | (0.446) | | -0.795(0.054) | | -0.694(0.067) | | (0.947) | | |
| % Change | (0.643) | | (0.459) | | (0.994) | | (0.482) | | (0.573) | | |
| **Total Power/Overall Autonomic Nervous System Activity** | | | | | | | | | | | |
| Before | 0.494(0.019) | | 3.387(0.053) | | -0.636(0.072) | | -0.618(0.055) | | (0.739) | | |
| After | 0.490(0.029) | | -3.414(0.054) | | -0.651(0.072) | | -0.769(0.020) | | (0.704) | | |
| % Change | (0.476) | | 22.644(0.064) | | (0.999) | | (0.681) | | (0.860) | | |
| **RMSSD/Parasympathetic** | | | | | | | | | | | |
| Before | (0.436) | | (0.463) | | (0.550) | | (0.246) | | (0.543) | | |
| After | (0.333) | | -0.011(0.033) | | (0.535) | | (0.120) | | (0.995) | | |
| % Change | (0.385) | | (0.780) | | (0.973) | | (0.802) | | 0.348(0.051) | | |
| **SDNN/ Overall Autonomic Nervous System Activity** | | | | | | | | | | | |
| Before | 14.940(0.015) | | (0.116) | | (0.220) | | (0.201) | | (0.657) | | |
| After | 14.505(0.013) | | 0.027(0.042) | | (0.115) | | -17.538(0.042) | | (0.858) | | |
| % Change | (0.306) | | (0.315) | | (0.699) | | (0.529) | | (0.899) | | |
| **Heart Rate (bpm)** | | | | | | | | | | | |
| Before | (0.199) | | (0.310) | | (0.200) | | 9.521(0.068) | | (0.266) | | |
| After | (0.173) | | (0.297) | | (0.495) | | (0.144) | | (0.416) | | |
| % Change | (0.474) | | (0.396) | | (0.139) | | (0.383) | | (0.557) | | |

**SUPPLEMENT Table 3: Anxiety Disorders and HRV Patterns**

| **Relation to Stressor** | **Anxiety** | | | | **Fear of Childbirth^*^** | | **Traumatic Events (>5)** | |
| --- | --- | --- | --- | --- | --- | --- | --- | --- |
|  | **State** | | **Trait** | |  |  |  |  |
| N (%) | 19.2 | | 31.4 | | 13.0 | | 35.2 | |
| **HF (P= Power, %= Percentage)/Vagal Modulation** | | | | | | |  | |
|  | P | % | P | % | P | % | P | % |
| Before | (0.161) | (0.332) | (0.483) | (0.663) | (0.121) | (0.353) | -0.683(0.14) | -10.9 (0.006) |
| After | (0.420) | (0.141) | (0.503) | -6.526  (0.078) | -0.563  (0.068) | (0.203) | -0.495(0.40) | (0.290) |
| % Change | (0.550) | (0.749) | (0.906) | -0.167  (0.078) | (0.706) | (0.497) | (0.151) | (0.105) |
| **LF (Power)/Sympathetic and Some Parasympathetic** | | | | | | |  | |
| Before | (0.301) | | (0.561) | | (0.202) | | (0.425) | |
| After | (0.651) | | 0.443(0.006) | | (0.341) | | (0.139) | |
| % Change | (0.247) | | 0.049(0.050) | | (0.924) | | (0.509) | |
| **LFHF Ratio/Sympathetic and Parasympathetic Ratio** | | | | | | |  | |
| Before | (0.498) | | (0.640) | | (0.279) | | 1.104 (0.004) | |
| After | (0.332) | | (0.273) | | 1.078(0.017) | | 222.509 (0.019) | |
| % Change | (0.696) | | 0.356(0.019) | | (0.552) | | (0.111) | |
| **VLF/Sympathetic and Renin-Angiotensin** | | | | | | |  | |
| Before | (0.474) | | (0.391) | | (0.357) | | (0.401) | |
| After | (0.693) | | (0.518) | | -0.519(0.044) | | (0.123) | |
| % Change | (0.966) | | (0.637) | | (0.180) | | (0.408) | |
| **Total Power/Overall Autonomic Nervous System Activity** | | | | | | |  | |
| Before | (0.112) | | (0.502) | | (0.348) | | -0.297 (0.079) | |
| After | (0.467) | | (0.371) | | -0.451(0.043) | | 0.354 (0.048) | |
| % Change | (0.539) | | (0.994) | | (0.339) | | (0.715) | |
| **RMSSD/Parasympathetic** | | | | | | |  | |
| Before | (0.109) | | (0.879) | | (0.512) | | (0.150) | |
| After | (0.365) | | (0.147) | | (0.778) | | (0.209) | |
| % Change | (0.145) | | (0.134) | | (0.438) | | (0.861) | |
| **SDNN/ Overall Autonomic Nervous System Activity** | | | | | | |  | |
| Before | -8.625(0.095) | | (0.494) | | (0.158) | | (0.122) | |
| After | (0.423) | | (0.380) | | -10.020(0.085) | | -8.874 (0.058) | |
| % Change | (0.703) | | (0.666) | | (0.295) | | (0.714) | |
| **Heart Rate (bpm)** | | | | | | |  | |
| Before | 5.632(0.056) | | (0.700) | | (0.251) | | (0.214) | |
| After | (0.166) | | (0.623) | | (0.222) | | (0.226) | |
| % Change | -0.024(0.092) | | (0.960) | | (0.517) | | (0.537) | |

**SUPPLEMENT Table 4: State and Trait Anxiety, Fear of Childbirth, and Exposure to Greater than Five Traumatic Events**

*Results for Delivery Fear are from a model with BMI included. BMI was only significantly associated with Fear of Childbirth of the mental health variables.

**Table 5: Brief Overview of HRV Measures**

| **Parameter** | **Unit** | **Description** | **Important Considerations** |
| --- | --- | --- | --- |
| HR | bpm | Heart rate, beats per minute |  |
| HRV Frequency-Domain Measures | | | |
| HF Frequency | Percentage | Relative power of the high-frequency band (0.15-0.4 Hz) | - Divides the absolute power for HF band by the summed absolute power of LF and HF bands - Compare despite individual variation |
| HF Power | ms^2^ | Absolute power of the high-frequency band (0.15-0.4 Hz) | - Also known as Respiratory Sinus Arrythmia (RSA) - PNS activity - Affected by breathing - Corresponds to heart rate variations related to respiration - Can be dissociated from vagal tone during large scale changes in SNS - Under controlled conditions while breathing at normal rates natural logarithm (ln) can estimate vagal tone |
| LF Power | ms^2^ | Absolute power of the low-frequency band (0.15-0.4 Hz) | - Reflects baroreceptor activity and blood pressure regulation during resting conditions - Both PNS and SNS, but more vagal mediation during deep breaths, lower respiration rates and less SNS |
| LF/HF Ratio |  | Ratio of LF-to-HF power | - Affected by breathing - May estimate the ratio between SNS and PNS under controlled conditions - Not a measure of SNS/PNS balance since PNS activity can be associated with decrease, increase, or no change in SNS activity |
| Total Power | ms^2^ | Sum of VLF, HF, and LF bands |  |
| VLF Power | ms^2^ | Absolute power of the very-low-frequency band (0.0033-0.04 Hz) | - Requires at least 5 minutes recording period - VLF is more associated with mortality - May be generated by physical activity, thermoregulatory influences, renin-angiotensin, and endothelial influences on the heart - PNS is contributory - Efferent SNS activity from physical activity and stress modulate amplitude and frequency |
| Time-Domain Measures | | | |
| RMSSD | ms | Root mean square of successive RR interval differences | - More representative of vagal mediation, more influenced by PNS - Correlated with HF power |
| SDNN | ms | Standard deviation of NN intervals | - Both SNS and PNS contribute, greater PNS in shorter readings and paced breathing - Correlated w/ VLF, LF, Total power |

Source: Adapted from Shaffer F and Ginsberg JP (2017) An Overview of Heart Rate Variability Metrics and Norms. *Frontiers in Public Health*, 5(258): 1-17.
